# An Infrared Absorbance Sensor for the Detection of Melanoma in Skin Biopsies

**DOI:** 10.3390/s16101659

**Published:** 2016-10-10

**Authors:** Valeria Fioravanti, Lukas Brandhoff, Sander van den Driesche, Heimo Breiteneder, Melitta Kitzwögerer, Christine Hafner, Michael J. Vellekoop

**Affiliations:** 1 Institute for Microsensors, Actuators and Systems (IMSAS), MCB, University of Bremen, Bremen D-28359, Germany; lbrandhoff@imsas.uni-bremen.de (L.B.); sdriesche@uni-bremen.de (S.v.d.D.); mvellekoop@imsas.uni-bremen.de (M.J.V.); 2 Department of Pathophysiology and Allergy Research, Medical University of Vienna, Vienna A-1090, Austria; heimo.breiteneder@meduniwien.ac.at (H.B.); christine.hafner@meduniwien.ac.at (C.H.); 3 Department of Pathology, University Hospital St. Poelten, Karl Landsteiner University of Health Sciences, St. Poelten A-3100, Austria; melitta.kitzwoegerer@stpoelten.lknoe.at; 4 Department of Dermatology, University Hospital St. Poelten, Karl Landsteiner University of Health Sciences, St. Poelten A-3100, Austria

**Keywords:** infrared sensor, absorbance spectroscopy, skin biopsy, melanoma

## Abstract

An infrared (IR) absorbance sensor has been designed, realized and tested with the aim of detecting malignant melanomas in human skin biopsies. The sensor has been designed to obtain fast measurements (80 s) of a biopsy using a small light spot (0.5 mm in diameter, typically five to 10 times smaller than the biopsy size) to investigate different biopsy areas. The sensor has been equipped with a monochromator to record the whole IR spectrum in the 3330–3570 nm wavelength range (where methylene and methyl stretching vibrations occur) for a qualitative spectral investigation. From the collected spectra, the CH_2_ stretch ratio values (ratio of the absorption intensities of the symmetric to asymmetric CH_2_ stretching peaks) are determined and studied as a cancer indicator. Melanoma areas exhibit different spectral shapes and significantly higher CH_2_ stretch ratios when compared to healthy skin. The results of the infrared investigation are compared with standard histology. This study shows that the IR sensor is a promising supportive tool to improve the diagnosis of melanoma during histopathological analysis, decreasing the risk of misdiagnosis.

## 1. Introduction

Cutaneous malignant melanoma is the deadliest and most aggressive form of skin cancer and is fatal if left untreated [1,2]. Cutaneous melanoma originates from melanocytes, pigment-producing cells located together with keratinocytes in the basal layer of the skin, close to the dermis [3]. Despite melanoma comprising only 4% of all skin cancers, it is responsible for 80% of skin cancer–related deaths [3,4]. The incidence of melanoma has been increasing over the past decades. In Europe, more than 100,000 new cases were estimated in 2012 [5].

The first step for diagnosis of melanoma is based on visual examination, i.e., assessing the lesion using the ABCDE approach, observation of abnormalities in asymmetry, border, color, diameter and evolution of the suspicious skin portion [6]. Most melanomas often show these features. If malignancy is suspected, the lesion is biopsied for histopathological examination that consists of a preliminary tissue staining followed by morphological inspection under the microscope. Histopathology is the gold standard for melanoma diagnosis; however, it remains a subjective method highly based on the expertise and training of the pathologist. Clinical diagnosis of melanoma is sometimes difficult also for expert pathologists since many benign lesions or common benign inflammatory conditions resemble malignancies upon visual investigation [1,7,8]. Hence, there is a need to develop new strategies for an accurate and precise diagnosis of melanoma.

The tremendous progress in optical, electronic and computational technology and instrumentation of the last decades has also encouraged the development of new, sophisticated approaches for melanoma diagnosis. Both in vivo and ex vivo strategies have been addressed, the first with the aim to evaluate suspicious lesions prior to excision, avoiding unnecessary biopsies, and the second to support clinical histopathology of biopsies, preventing the risk of false positive and false negative results. Techniques such as dermoscopy, confocal laser scanning microscopy (CLSM), optical coherence tomography (OCT), infrared thermography, electrical impedance, and vibrational spectroscopy have been tested for detection of melanoma [9,10].

Dermoscopy involves the use of a handheld microscope with a magnification lens and polarized light to examine the suspect lesion down to the deeper dermis structure not visible to the human eye. Although dermoscopy has had the largest clinical impact as an emerging technology for skin cancer diagnosis, it is not a diagnostic tool and it is strongly dependent on the expertise of the operator [11]. In vivo, non-invasive CSLM, acquiring in-focus images at different depths for 3D reconstruction imaging, has shown remarkable potential in identification of malignant melanomas [12,13]. Its high cost and strong-contrast attenuation due to hyperpigmented or hyperkeratoic lesions represent the main limitations of this technique [14]. OCT is another in vivo, non-invasive technique using scattered coherent light from the tissue to create images where contrast is due to different reflectivity of the tissue components. Gamblicher et al. used OCT to investigate malignant melanoma with quite promising results [15]. However, the utility of OCT in skin lesion diagnosis has not been established yet and accuracy studies are still missing [16]. Infrared thermography can be used to detect the local higher warmth developed by cancerous lesions that are metabolically more active than healthy tissue [17]. Infrared thermography records the radiation emitted by the human body with no need for external illumination. Attempts to localize cutaneous melanomas by infrared thermography have been made by several research groups [18,19,20]. However, infrared thermography does not permit us to distinguish between different kinds of skin cancers and requires careful calibration for the correct assignment of temperature to emitted radiation [9]. Other methods based on evaluation of skin electrical impedance have been proposed and tested for detection of malignant lesions [21,22]. Scibase [23] have developed an electrical impedance spectroscopy (EIS) product, Nevisense^TM^, applying a specific range of frequencies to the skin to extract information about different cellular properties of the lesion under analysis. This method was demonstrated to improve accuracy in melanoma diagnosis, reducing the number of unnecessary biopsies [24]. However, the use of Nevisense^TM^ is limited to specific body sites and to lesions fulfilling specific criteria [25]. Vibrational spectroscopy has also been considered as a potential tool for tissue screening [26,27,28]. Several relevant studies have reported the potential of vibrational spectroscopies, infrared absorption, Raman scattering and their multiple derivative techniques to identify and characterize skin molecular structures as well as to discriminate several skin lesions [29,30]. Many works employed the use of computer-based multivariate statistical analysis (MSA) techniques to extract subtle spectral differences from the acquired infrared spectra. Concerning melanoma detection and screening, several works for in vivo and ex vivo measurements by using middle-, near-infrared, Fourier transform infrared spectroscopy (FTIR) and Raman spectroscopy have been published [31,32,33]. However, spectroscopy techniques such as FTIR or Raman are still far from being routinely applied for diagnostic purposes since their high cost and complexity make them inaccessible to hospitals or smaller practices. Recently, broadly tunable quantum cascade lasers (QCLs) have been successfully integrated and employed for spectroscopy and its applications in order to overcome limitations of current spectroscopy instrumentation [34,35,36,37]. Due to their higher spectral power density and high brilliance, QCLs do not require the use of interferometers, leading to simpler instrument setup [38]. Moreover, the high brightness of QCLs allows the use of room-temperature microbolometer detector systems, with no need for cryogenically cooled detectors [36]. Despite the undeniable potential of this technology, current studies are focused on demonstrating its feasibility in the clinical setting, especially regarding the image quality and signal-to-noise ratio [39]. Moreover, QCLs remain expensive infrared source solutions whose room-temperature wavelength operation range has been extended only very recently to the 3–4 µm range [40].

Despite the efforts to develop new methods and technologies, a more accurate diagnosis method for the inspection of melanoma has not been established yet.

In previous works, we studied the possibility of using the CH_2_ stretch ratio (i.e., the ratio of the symmetric and asymmetric CH_2_ peak absorptions at wavelengths of 3505 nm and 3420 nm, after baseline correction) as a cancer indicator. Indeed, anomalies in the infrared absorption of methylene (CH_2_) groups have been reported in the literature in the occurrence of cancer [41,42]. We realized an infrared quadruple sensor for the CH_2_ stretch ratio determination of different melanoma cell lines [43,44]. Increased CH_2_ stretch ratio values were observed for melanoma cells compared to the healthy reference. However, the application of the CH_2_ stretch ratio method to the analysis of skin tissues demanded the realization of a new infrared sensor due to the higher biological complexity of tissues compared to cell lines.

In this work, a label-free IR sensor system for detection of melanoma in human skin biopsies is proposed. The IR sensor records absorption spectra (in the range of 3330–3570 nm) of biopsies in 80 s. The IR light spot 0.5 mm in diameter permits the analysis of several regions of a biopsy (typical size 3–5 mm in diameter). That is an advantage when different suspicious areas within a single biopsy need to be investigated. A MATLAB script controls data acquisition, processing and storage and determines the CH_2_ stretch ratio values in each biopsy spot. The aim of this work is to investigate whether the IR sensor can be used as a complementary tool to standard histopathological inspection.

After histological examination by a pathologist, several human skin biopsies including healthy skin tissue, melanomas and melanoma metastasis have been investigated with the IR sensor. The sensor outcomes have been compared and related to the histological results.

## 2. Materials and Methods

### 2.1. CH_2_ Stretch Ratio as a Cancer Indicator

Abnormalities in the infrared spectrum of skin lesions have been registered when carcinogenesis occurs [41,42]. Instead of recording the whole infrared spectrum (1–20 µm) to monitor changes in absorption due to cancer presence, we use an infrared absorbance method focused in the restricted IR region between 3330 nm and 3570 nm (2800–3003 cm^−1^). This spectral range is characterized by the stretching vibrational modes from methyl and methylene groups, mainly associated with lipid alkyl/acyl chains. In particular, two strong absorption bands due to the lipid methylene asymmetric and symmetric stretching modes arise at 3420 nm (2923 cm^−1^) and 3505 nm (2853 cm^−1^), respectively. The change in the CH_2_ stretch ratio can be explained with disorders in the lipid chains of membrane, resulting in modified lipid packing, induced by cancer occurrence. These disorders lead to higher CH_2_ symmetric stretching modes and consequently to higher CH_2_ stretch ratio [45].

**Figure 1:**
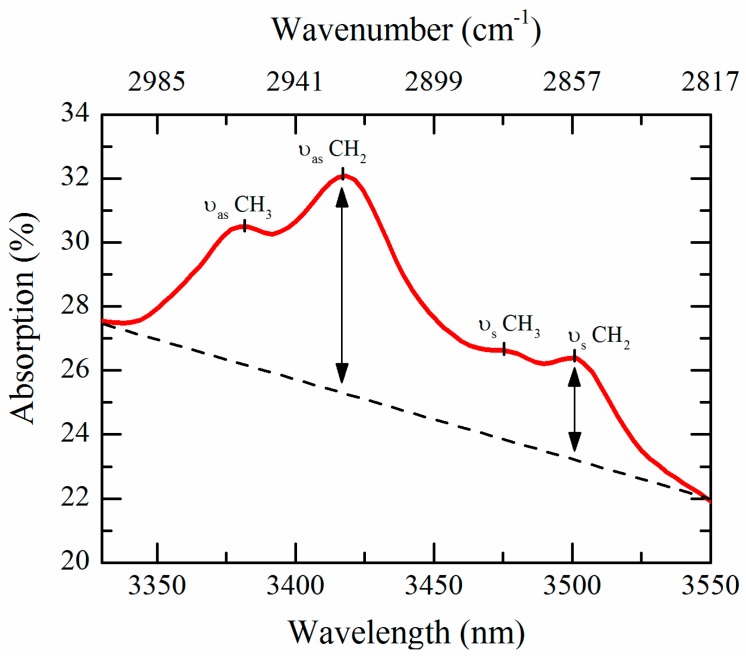
Typical infrared absorption spectrum of healthy skin tissue in the 3330–3570 nm wavelength range (2800–3003 cm^−1^) acquired using a Bruker Hyperion 3000 IR microscope coupled to a Tensor 37 spectrometer. The spectrum was recorded at 4 cm^−1^ resolution for a total of four scans. Methyl (CH_3_) and methylene (CH_2_) symmetric (ν_s_) and asymmetric (ν_as_) stretching modes are indicated. The CH_2_ stretch ratio is computed from the ratio between the CH_2_ symmetric peak at 3505 nm and the CH_2_ asymmetric peak at 3420 nm after straight baseline subtraction.

As shown in Figure 1, the CH_2_ stretch ratio is calculated from the height of the CH_2_ vibration peaks after linear baseline correction using two further wavelengths as reference points (3335 and 3550 nm). Only these four functional wavelengths are needed for the calculation of the CH_2_ stretch ratio. The spectrum of the empty calcium fluoride (CaF_2_) slide (26 × 65 × 1 mm^3^) used as substrate is acquired and subtracted from the biopsy spectrum. CaF_2_ has been chosen as substrate material for its high transparency in the mid-infrared range of interest (>92% in the 3330–3570 nm spectral range).

### 2.2. Infrared Absorbance Sensor

Infrared optical investigation usually requires expensive and complex instruments covering the whole infrared range (1–20 µm) such as Fourier transform infrared (FTIR) spectroscopy or a Raman setup. Despite their high sensitivity and resolution, these technologies are relatively complex or expensive. The IR sensor used for an objective melanoma detection method is a portable, cost-effective, easy-to-handle instrument.

**Figure 2:**
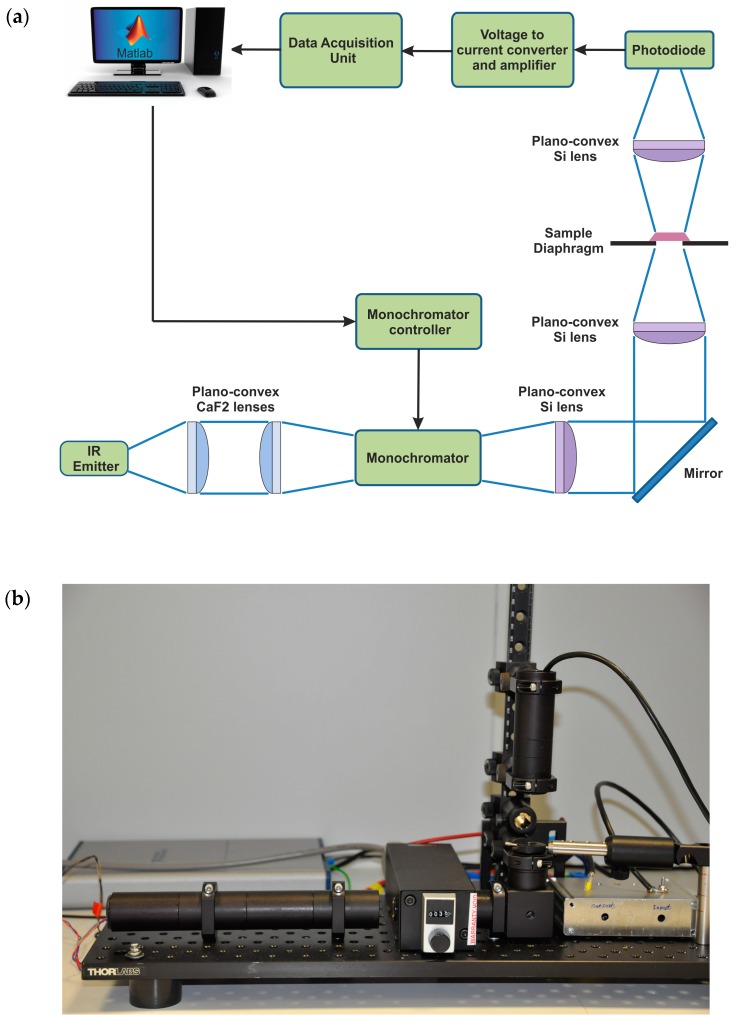
(**a**) Schematic of the overall IR instrument setup. The system includes a thermal emitter as infrared light source, a monochromator for light filtering, an optical lens system to guide the light, and a photodiode for signal detection. The light beam is collimated and focused into the entrance of the monochromator by plano-convex CaF_2_ lenses (f = 75 mm). After the monochromator, the light is collimated by a Si plano-convex lens (f = 25 mm) and vertically directed by an IR mirror. A second Si plano-convex lens focuses the light beam into the sample holder where the specimen is placed for IR analysis. The light transmitted by the sample is collected by a third Si plano-convex lens and focused onto the active area of the photodiode; (**b**) Photo of the IR sensor system.

A schematic of the overall system and a picture of the IR sensor are shown in Figure 2a,b, respectively. A straight-forward infrared technology (infrared emitter as light source, photodiode as detector, monochromator for light filtering) has been preferred to expensive infrared lasers and nitrogen cooled infrared detectors, lowering the realization costs and the maintenance costs of the system. We used a micro-machined thermal emitter from HawkEye Technologies, Milford, CT, USA. The infrared light source is set to operate at 5 Hz (50% duty cycle) to allow several measurements per second. According to the light source technical specification, the use of higher operation frequencies would lower the modulation depth and consequently the intensity of the emitted light pulses. A monochromator from Optometric Corporation, USA is used to disperse single wavelengths from the polychromatic light emitted by infrared source. It operates in Fastie-Ebert configuration consisting of a single large spherical mirror and one plane diffraction grating. The entrance slits of the monochromator are 0.3 × 4 mm^2^ in size. The optical resolution of the monochromator for the filtered light is 12 nm, which is sufficient for our measurement method. The stepper motor of the monochromator is connected to a microcontroller DCB-241 from Advanced Micro Systems (AMS) (Liberty Hill, TX, USA). The monochromator filters out 80 single wavelengths with 3 nm steps in the infrared range between 3330 nm and 3570 nm. Rotation of the step motor occurs in 0.1 s. In this time, no data acquisition is performed. The optical system guiding the light inside and outside the monochromator critically depends on the f number of the monochromator. To ensure the maximum wavelength accuracy and system throughput, the effective aperture of the beam entering the monochromator needed to be f/3.9 or greater, with f the focal length of the monochromator (74 mm). Therefore, light must be focused on the entrance slit of the monochromator using a lens with comparable f number. For this reason, two CaF_2_ lenses from Thorlabs GmbH, Munich, Germany (LA5042) of 75 mm focal length are used to collimate and focus the light at the entrance slit of the monochromator. Two 25 mm focal length Silicon (Si) lenses from Thorlabs GmbH, Munich, Germany (LA8281) and a mirror are used to focus the light on the sample holder. A diaphragm with adjustable diameter size is placed in the sample holder to regulate the spot size of the light illuminating the sample.

The spot size has been adjusted to 0.5 mm in diameter in order to investigate small, specific regions of a skin biopsy. The light transmitted by the sample is detected by an InAs photodiode from IoffeLED (St. Petersburg, Russia) operating at room temperature. Among the photodiodes available in the market, this photodiode provides a good compromise between cost and performance in the spectral range of interest. With a peak wavelength detection at 3305 nm and a cutoff wavelength at 3700 nm, it has a good sensitivity (>1 A/W) in the range 3330 nm–3570 nm. A three-stage amplifier with build-in low-pass filtering is used for amplification of the photodiode signal. An AD797 (Analog Devices, Norwood, MA, USA) based trans-impedance-amplifier converts the current from the photodiode into a voltage signal. The trans-impedance gain is set via feedback to 10 kV/A. The signal is filtered by a single RC-lowpass filter with a bandwidth of 300 kHz, and amplified 18 times in two more stages using an operational amplifier AD8599 (Analog Devices, Norwood, MA, USA). This results in a total gain of the system of 180 kV/A.

The wavelengths filtered by the monochromator are sampled with a rate of 150 kHz and an average voltage value is extracted for each wavelength (80 in total).

**Figure A1:**
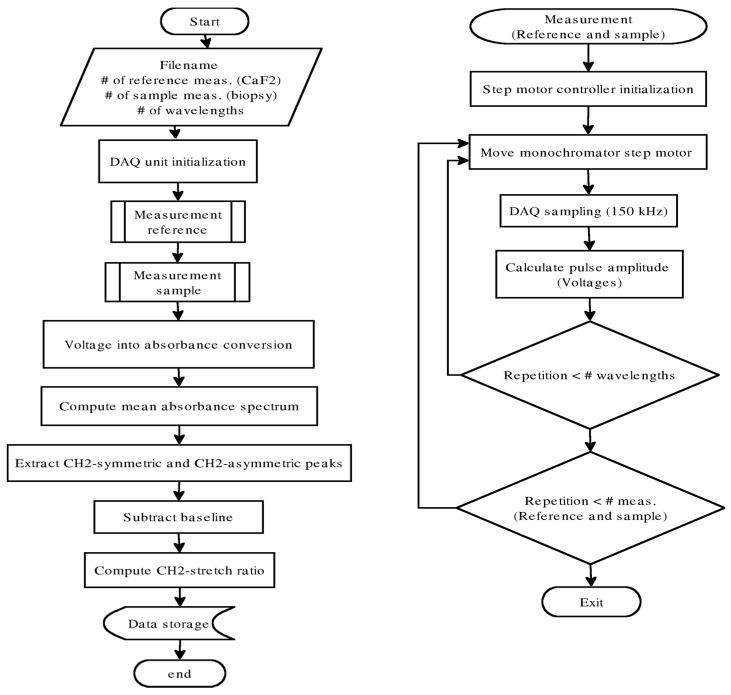
Flowchart of the MATLAB script structure. The flowchart is organized in two processes. The main process sets the user defined parameters, initializes the DAQ unit, performs the measurements, determines the IR absorbance spectrum and computes the CH_2_ stretch ratio. The second process represents the measurement process driving the step motor of the monochromator, evaluating the pulse amplitude in voltage for each wavelength and for the user defined number of repetitions.

A MATLAB script controls the step motor of the monochromator, reads out the amplified photodiode signal, extracts infrared absorbance spectra and derives the CH_2_ stretch ratio from the biopsy samples. The number and the speed of the measurements along with the sampling rate can be adjusted by the user in the MATLAB user interface. In standard operation conditions, the software script commands the step motor of the monochromator to move every second to the next wavelength (3 nm steps). Five measurements are made within this one second, according to the frequency of the light source (5 Hz). The data voltages acquired for each wavelength are averaged and then converted into absorbance values according to Beer-Lambert law and plotted in the infrared absorbance spectrum. A detailed flowchart of the MATLAB script has been shown in Figure A1 to explain the algorithm logic.

From the obtained spectrum, the CH_2_ stretch ratio value is calculated as described in Section 2.1. The small size of the infrared light spot (0.5 mm in diameter) permits to extract spectral information and CH_2_ stretch ratio values from multiple spots of the biopsy under analysis. This is of great help when biopsy with different morphological features needs to be investigated.

### 2.3. Skin Biopsy Preparation for IR Analysis

Samples of primary melanomas and metastasis were taken from lesions with a diameter of over 1 cm. Normal skin samples were collected to be used as reference for the infrared measurements. All the specimens were obtained by punch biopsy during standard surgeries immediately after removing the surgical specimen. The biopsy samples were immediately frozen in liquid nitrogen and stored at −70 °C. The surgical specimen was submitted to routine histopathological examination. For each biopsy sample, five 20-µm-thick serial sections were cut. Three sections were fixed on a glass slide for hematoxylin and eosin (H&E) staining and histological diagnosis by a dermatopathologist. The other two sections were fixed with a drop of distilled water on an infrared transparent calcium fluoride (CaF_2_) slide for IR absorbance measurements. The study was approved by the local ethical committee of Lower Austria (GS4-EK-3/063-2011).

## 3. Results

For this study, 15 human skin biopsies were analyzed, including samples of healthy skin, nodular melanomas, melanoma metastases and superficial spreading melanomas. Standard histological examination was performed by an expert histopathologist prior to the infrared investigation by the IR sensor.

**Figure 3:**
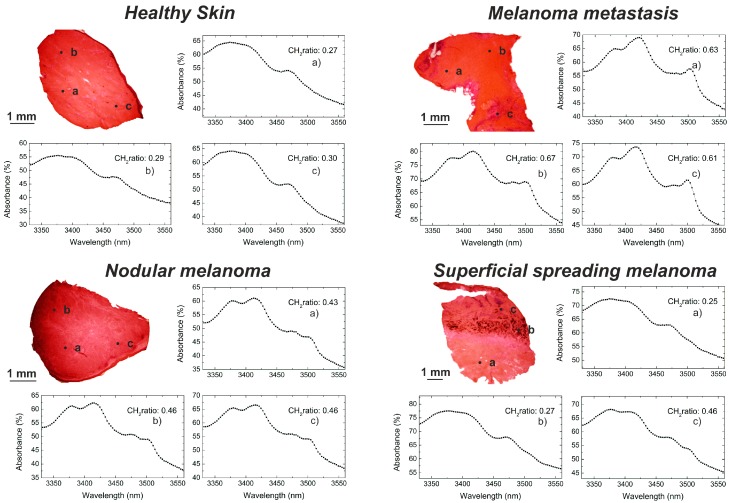
H&E stained pictures of healthy skin tissue, nodular malignant melanoma, superficial spreading malignant melanoma and melanoma metastasis investigated by the IR sensor. Infrared spectral information and CH_2_ stretch ratio values have been obtained from three different biopsy spots. The plotted infrared spectra originate from a sample area of ~0.5 mm in diameter. Spectral shapes of healthy and cancerous tissue exhibit significant differences. The CH_2_ stretch ratio values of malignant melanomas and melanoma metastasis are increased compared to the healthy tissue of reference.

H&E pictures of a subset of samples (including one of each kind of biopsy) are shown in Figure 3. The biopsy spots where infrared measurements have been performed are indicated by letters (a, b, c) for each sample. Histologic results for the healthy skin show regular epidermis with a hyperpigmented basal layer. No tracks of cancerous cells have been diagnosed. The examination of the nodular melanoma revealed a Breslow thickness (penetration depth of the tumor) of 15.0 mm with no ulceration. Sheets of polymorphous melanoma cells infiltrate the whole dermis. In the epidermis only sparse infiltration of tumor cells can be detected together with signs of subepidermal regression. Melanoma cells are homogeneously spread over the whole biopsy section. In the melanoma metastasis the pathologist recognized the presence of extended areas of necrotic melanoma cells in the subcutaneous tissue and skeletal muscle. The superficial spreading melanoma shows ulceration and a Breslow thickness of 3.0 mm. The ulcerated tumor has invaded the deep dermis with prominent pigment formation (a) followed by a region of tumor-free subcutaneous tissue (b). Extended regions of inflammatory cells are present in the dermis (c).

After histological examination, the biopsies have been disposed for infrared measurements using the IR sensor. Absorbance infrared spectra around the C-H stretching region were collected from each biopsy in three different spots to detect eventual morphological changes within a single biopsy. Each biopsy spot was measured five times and the plotted infrared spectra originate from the average of those five spectra.

Except for the superficial spreading melanoma, the analyzed biopsies appear internally quite homogenous, with similar infrared responses over the three investigated spots. This is in line with the findings from the histopathologist who recognized a fully healthy area for the normal skin and extended melanoma regions for the nodular melanoma and the melanoma metastasis. Different outcomes were obtained from the infrared investigation of the superficial spreading melanoma, where the infrared responses of the healthy subcutaneous area and the inflammatory cells (highly comparable) exhibit significant differences compared to the melanoma-invaded region.

Comparing healthy and tumor tissues, different spectral shapes and CH_2_ stretch ratios can be noticed. Qualitatively, a strong signal from methyl-containing molecules can be noticed in healthy skin tissues compared to melanomas. Both the asymmetric and symmetric stretching modes of methyl groups are increased compared to cancerous tissue, indicating an abundance of methyl groups. In case of melanomas, the 3330–3570 nm region is dominated by the strong absorption bands of asymmetric and symmetric stretching modes of the alkyl/acyl chains around 3420 and 3505 nm. Weaker bands due to the asymmetric and symmetric stretching modes of methyl groups are observed around 3380 nm and 3480 nm. These spectra are a characteristic feature of samples with an abundance of acylglycerolipids and sphingolipids [33].

From the collected infrared spectra, the CH_2_ stretch ratio values were computed, as described above. The CH_2_ stretch ratio values significantly increase in the occurrence of cancer. From values between 0.27 and 0.30 obtained in cases of normal skin tissues, the CH_2_ stretch ratios increase up to 0.43 and 0.46 for the melanomas. The melanoma metastases are characterized by even higher CH_2_ stretch ratio values between 0.61 and 0.67. The infrared investigation of the superficial spreading melanoma has been conducted, focusing on the healthy skin subcutaneous tissue, the inflammatory region and the tumor area.

It can be noticed that the responses from the subcutaneous tissue and the inflammatory cells show the same spectral characteristics of the previously measured healthy skin tissues, with a mean CH_2_ stretch ratio between 0.25 and 0.27. Methyl-rich areas contribute to enhancing both the CH_3_ asymmetric and symmetric bands compared to cancerous tissue. The CH_2_ stretch ratio calculated for the tumor area gives a higher value of 0.46.

## 4. Discussion

The discrepancy between the absorption responses of healthy and cancerous tissues might be due either to different molecular components or to different organization of the acyl chains in membrane lipids or to conformational alterations in the acyl chains themselves [45]. Indeed, the more a chain is disordered, with consequent generation of kinks and twists, the higher the CH_2_ symmetric stretch absorbance is [46], leading to a higher CH_2_ stretch ratio. Furthermore, many studies highlighted that carcinogenesis is combined with alterations in the plasma membrane due to a reduction of cholesterol concentration [47]. Cholesterol is normally acting as a stabilizing agent in the lipid membrane. In the case of cancer, a reduction in the cholesterol concentration leads to a molecular disorder with a consequent increase of the CH_2_ symmetric stretch vibration [48]. Our previous studies on melanoma cell lines showed an increase in the CH_2_ stretch ratio when cholesterol is sequestered from the plasma membrane [43].

As mentioned, the total measurement time for one spot in a biopsy is about 80 s. Further improvements of the measurement speed of the system can be achieved by using a continuous wave (CW) IR light source instead of the pulsed source. The reference measurement (light switched off) will be acquired only one time at the beginning of the measurement. The gain in the measurement time can be more than a factor of two, allowing us to analyze a biopsy spot in less than 30 s. This will be implemented in our system.

An even smaller light spot size could be obtained by coupling the IR light source into an IR-transparent optical fiber. However, the technical specifications of the used IR light source do not permit us to obtain an optimal coupling efficiency to the fiber due to the loss of a consistent portion of the incoming light.

As discussed, the absorbance value at a single wavelength is derived in 1 s after the acquisition of 150,000 voltage values. The spectra plotted in Figure 3 result from averaging five spectra in the same biopsy spot. Five-time spectral averaging has been considered for our experiments as a good tradeoff between quality of the spectra and reduced measurement time, with no need to use denoising algorithms. Although a higher number of averaging would be advantageous to decrease the random noise and improve the spectral results, this would be obtained at the cost of longer data acquisition.

## 5. Conclusions

We presented an efficient and effective label-free infrared sensor to support pathologists during the critical diagnostic phase of skin biopsies. The small IR light spot, the measurement speed and the IR spectral acquisition allow fast and accurate inspection of a skin biopsy.

The versatile user interface in MATLAB permits us to set the sensor parameters according to the user’s needs, allowing control of the amount and speed of the measurements and setting of the spectral measurement range. The spectral profiles in the C-H stretching region and the evaluated CH_2_ stretch ratio values of the examined samples are automatically returned at the end of the measurements. The results are obtained in 80 s.

Differences in the spectral responses of healthy skin, melanoma and metastasis biopsies were found. The measurements showed an increase of the CH_2_ stretch ratio values in the presence of cancer. Even melanocytic cluster regions and areas of inflammatory cell areas within one biopsy could be analyzed thanks to the small size of the infrared light spot. The inflammatory status did not show alterations of the CH_2_ stretch ratio compared to the healthy reference.

From these analyses, we can conclude that the sensor is a very promising instrument for detecting melanoma cancer in skin biopsies. The method can have a strong impact on improving the diagnosis in routine pathology, resulting in the further reduction of misdiagnosis.
